# T-bet-mediated differentiation of the activated CD8^+^ T cell

**DOI:** 10.1002/eji.201040873

**Published:** 2010-11-19

**Authors:** Crystal J J Yeo, Douglas T Fearon

**Affiliations:** Wellcome Trust Immunology Unit, Department of Medicine, University of Cambridge, Medical Research Council CentreCambridge, UK

**Keywords:** B lymphocyte-induced maturation protein 1, IL-2, IL-15 receptor α, T-box expressed in T cells

## Abstract

The T-box transcription factor, T-bet promotes the differentiation of short-lived effector CD8^+^ T cells at the expense of central memory cells. How T-bet mediates these effects, and whether they are directly caused by T-bet alone are unknown, because expression of T-bet requires stimulation of the T cell by inflammatory and growth cytokines, which may have T-bet-independent functions involving T-cell differentiation. We developed an *in vitro* system of ectopic T-bet expression that avoids the effects of inflammatory cytokines to determine which aspects of the T-bet phenotype may be accounted for by T-bet alone. Ectopic T-bet expression by OT-I CD8^+^ T cells stimulated by the H2-Kb (SIINFEKL) complex and cultured with 2 ng/mL IL-2 induced a coordinated change in gene expression leading to down-regulation of CD127 and SOCS-1 and up-regulation of CD122 and IL-15 receptor α, switching the cellular survival cytokine from IL-7 to IL-15. T-bet expression and 2 ng/mL IL-2 also led to a capacity for IFN-γ and Fas ligand expression, confirming a role in eliciting these effector functions. Finally, ectopic T-bet promoted the expression of B lymphocyte-induced maturation protein 1 by OT-I cells in the presence of 20 ng/mL IL-2, providing a mechanism for the role of T-bet in driving terminal differentiation in concert with a high level of IL-2 receptor signalling.

## Introduction

The T-box transcription factor T-bet was first described as the transcription factor that is responsible for the development of Th1 CD4^+^ T cells [1]. Since that report, T-bet has been found to be involved in the differentiation of several other types of immune cells, including macrophages [2], NK cells [3] and CD8^+^ T cells [4]. Although its role in the CD8^+^ T cell was initially thought to be non-essential [5], studies have recently shown that T-bet is involved in the differentiation of short-lived effector CD8^+^ T cells [6] at the expense of central memory cells [7], and to account for the defect in the development of central memory CD8^+^ T cells that occurs in CD4^+^ T-cell-deficient mice with viral infections [7]. Some of the phenotypic aspects that distinguish effector/effector memory CD8^+^ T cells from central memory cells that have been ascribed to T-bet are low expression of IL-7Rα, increased responsiveness to IL-15, and a short lifespan during acute viral infections [6]. However, which of these responses of the CD8^+^ T cell are a direct consequence of T-bet alone or of T-bet acting in concert with other factors, such as cytokines or other transcription factors, is not known, mainly because the studies of T-bet function in the CD8^+^ T cell have been conducted *in vivo* in which immunological stimuli cannot be easily controlled.

In considering additional signalling pathways that may interact with T-bet, it is notable that some phenotypes of the activated CD8^+^ T cell that have been ascribed to T-bet have also been associated with IL-2 receptor (IL-2R) signalling [8, 9] and with expression of the zinc finger-containing transcriptional repressor, B-lymphocyte-induced maturation protein 1 (Blimp-1) [10, 11]. For example, two recent studies comparing WT and IL-2Rα-deficient CD8^+^ T cells concluded that sustained IL-2R signalling during an acute viral infection led to the development of terminally differentiated effector cells in preference to central memory cells [8, 9], which is reminiscent of the phenotype associated with high T-bet expression [7]. Blimp-1, which may be a master regulator of the terminal differentiation of T cells [10, 11] as it is in B cells [12], is required for normal development of effector CD8^+^ T cells [13, 14], and its absence prevents the occurrence of “exhausted” CD8^+^ T cells [15] that have a phenotype similar to that of T-bet-dependent, “unhelped” CD8^+^ T cells [7]. Moreover, the finding that IL-2R signalling induces expression of Blimp-1 in the CD8^+^ T cell [16] may account for these two factors being associated with similar *in vivo* phenotypes of an antigen-stimulated CD8^+^ T cell, but this report did not evaluate the possible involvement of T-bet.

In this study, we developed an *in vitro* model system to determine those aspects of the T-bet phenotype that may be accounted for by T-bet alone, and whether T-bet links IL-2R signalling to the expression of Blimp-1.

## Results

**Figure 1:**
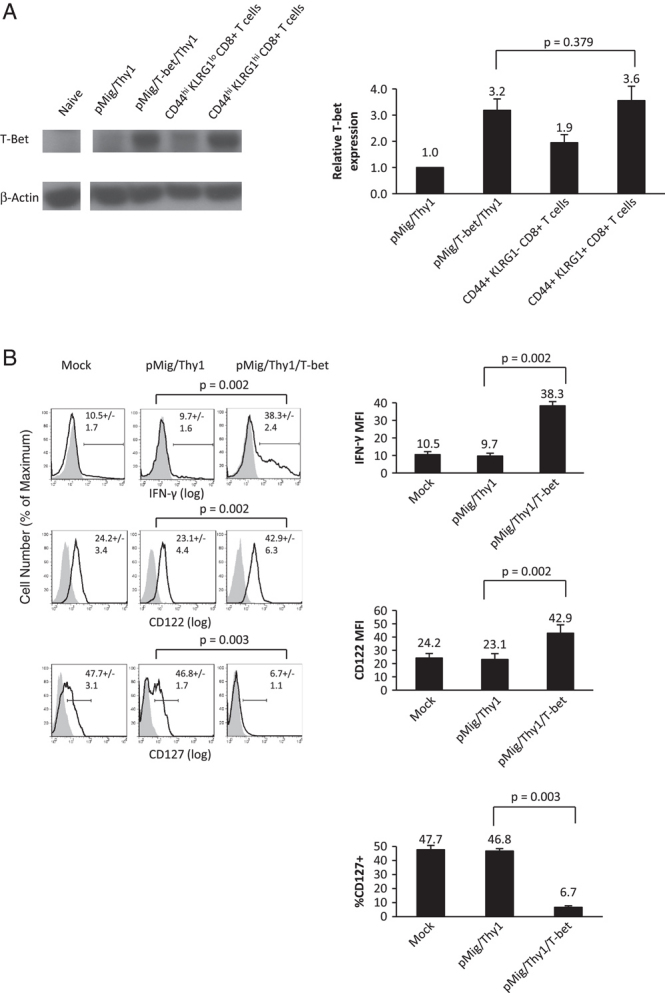
Ectopic expression of physiological levels of T-bet in CD8^+^ T cells. (A) OT-I cells that had been activated with 10 nM SIINFEKL (OVA) peptide in the presence of 2 ng/mL IL-2 and neutralising antibodies to IFN-γ, IFN-γR1 and IL-12 were held in buffer or transduced with pMig/Thy1 or pMig/Thy1/T-bet. On day 2 post-transduction, rat Thy1^+^ cells were MACS-isolated to greater than 98% purity and whole-cell lysates were prepared. Additionally, whole-cell lysates were prepared from CD44^hi^ KLRG1^lo^ and CD44^hi^ KLRG1^hi^ CD8^+^ T cells that had been purified from the spleens of three to four mice 10–12 wk post-infection with MHV-68. Naïve OT-I cells were used as a negative control for T-bet expression. The expression of T-bet and β-actin were measured three times for each determination using ImageJ. The means and SD of relative T-bet expression are shown. Results are representative of two independent experiments. Briefly, *p*-value was calculated using a two-tailed unpaired Student's *t*-test. (B) OT-I cells were transduced with the pMig retroviruses. On day 2 post-transduction, rat Thy1^+^ cells were evaluated for the expression of CD122 and CD127 by FACS analysis. In addition, cells were stimulated with SIINFEKL peptide, 2 ng/mL IL-2 and Brefeldin A for 3 h and evaluated for intracellular IFN-γ. The grey areas represent isotype staining and the black lines represent specific antibody staining. The MFI (CD122) and % cells specifically staining with anti-IFN-γ and anti-CD127 are represented by the numbers in the FACS plots and histograms. Cumulative means and SE from three to four independent experiments are presented. Briefly, *p*-values were calculated using two-tailed paired Student's *t*-tests.

**Figure 2:**
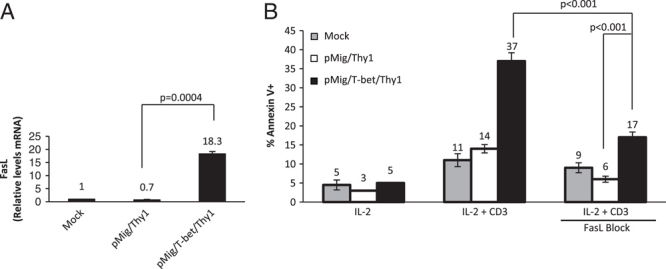
Ectopic expression of T-bet in CD8^+^ T cells and effects on FasL expression and activation-induced cell death. (A) OT-I cells were transduced and purified on day 2 post-transduction. RNA was extracted and assessed for FasL mRNA by qRT-PCR relative to CD3ɛ mRNA levels. All RNA samples were done in triplicate and the “No RT” controls were negative. The means and SD are shown for each determination. Results are representative of two independent experiments. Briefly, *p*-value was calculated using a two-tailed unpaired Student's *t*-test. (B) OT-I cells were transduced, purified and cultured in the presence or absence of plate-bound anti-CD3, IL-2 (2 ng/mL) and anti-IFN-γ antibodies. Anti-FasL antibody (20 μg/mL) was added to some cells to block ligation of Fas. After 20 h, cells were assessed for apoptosis by flow cytometry, following staining with Annexin V and 7-AAD. The percentage of Annexin-V expressing cells was determined in the viable 7-AAD-negative population. The means and SD are shown for each determination and results are representative of two independent experiments. Briefly, *p*-values were determined by a one-way analysis of variance followed by Tukey's multiple comparison test for comparisons between all pairs of conditions.

We examined the effects of T-bet that was ectopically expressed by retroviral transduction of OT-I CD8^+^ T cells grown in the presence of neutralising antibodies to IFN-γ and IL-12 [17], which are involved in endogenous T-bet expression, to enable an analysis of T-bet-mediated developmental changes that are independent of the effects of these two inflammatory cytokines. We also controlled the concentration of IL-2 by addition of defined amounts to CD8^+^ T cells that had been activated by antigen in the absence of CD28 co-stimulation, which would avoid cellular biosynthesis of this cytokine. By this means, we were able to generate two populations of OT-I cells that had identical exposures to antigen and cytokines, and differed only in their relative expression of T-bet (Fig. 1A). Moreover, the level of T-bet protein in the OT-I cells transduced with the T-bet-expressing retroviral vector was similar to that of highly differentiated, KLRG1^+^CD8^+^ T cells from mice persistently infected with murine gammaherpesvirus 68 (MHV-68) (Fig. 1A). Therefore, the finding that these T-bet^+^ OT-I cells maintained in culture by the presence of low amounts of IL-2 (2 ng/mL) differed from the control T-bet cells by increased expression of CD122 and almost absent expression of CD127 (Fig. 1B) indicates that this transcription factor is sufficient for these aspects of an effector CD8^+^ T cell. In addition to its promoting the effector function of TCR-inducible IFN-γ (Fig. 1B), ectopic T-bet also caused the expression of Fas ligand (FasL) (Fig. 2A), which was functional in that the T-bet^+^ OT-I cells mediated FasL-dependent activation-induced cell death (Fig. 2B). Thus, T-bet and a low level of IL-2R signalling are sufficient for these aspects of effector differentiation.

**Figure 3:**
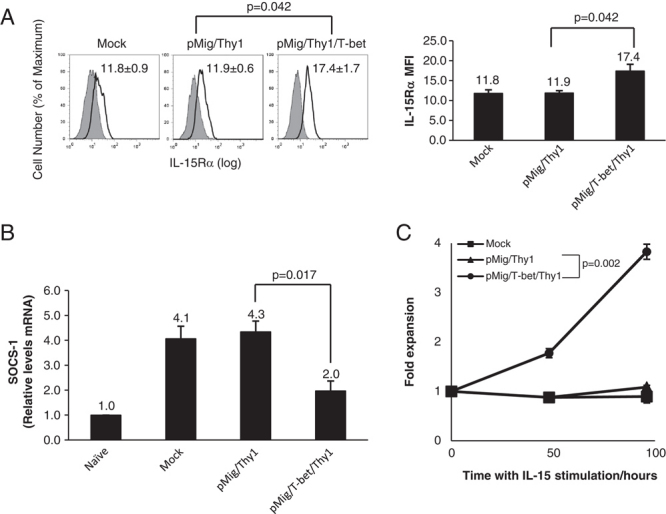
Ectopic expression of T-bet in CD8^+^ T cells and regulation of IL-15R signalling. (A) OT-I cells were transduced and purified. On day 2 post-transduction, Thy1^+^ cells were evaluated for IL-15Rα expression by FACS analysis. The grey area represents isotype staining and the black line represents specific antibody staining. The MFI for specific staining is represented by the numbers in each histogram. Cumulative means and SE of the MFI of specific IL-15Rα staining from three independent experiments are presented in the FACS plots and histograms. Briefly, *p*-value was calculated using two-tailed paired Student's *t*-test. (B) OT-I cells were transduced and Thy1^+^ cells assessed for SOCS1 mRNA by qRT-PCR relative to CD3ɛ mRNA levels. All RNA samples were done in triplicate and the “No RT” controls were negative. Cumulative means and SD from three independent experiments are shown. Briefly, *p*-value was calculated using two-tailed paired Student's *t*-test. (C) OT-I cells were transduced, purified and plated at 5×10^5^ cells/well with 20 ng/mL IL-15 in the presence of anti-IFN-γ antibodies. Viable cells were counted before plating and after 48 and 96 h. The means and SD are shown for each determination and results are representative of two or more independent experiments. Briefly, *p*-value was calculated at 96 h using two-tailed unpaired Student's *t*-test.

The down-regulation of IL-7Rα by T-bet resembles the phenotype of the “short-lived effector CD8^+^ T cell” [6], and an alternative means for long-term viability *in vivo* for such a cell would be signalling *via* the IL-15 receptor (IL-15R). In addition to confirming the known ability of T-bet to induce CD122 [6, 18], the IL-2/IL-15Rβ subunit, we examined two other elements controlling the IL-2/15R pathway: IL-15Rα and SOCS-1, which is a repressor of signalling by receptors containing the γ c chain. Analysis of T-bet^+^ and T-bet^−^ OT-I cells revealed that the former cells had increased IL-15Rα expression (Fig. 3A), and a two-fold reduction in SOCS-1 mRNA (Fig. 3B). These three relatively modest changes in the expression of IL-2/15Rβ, IL-15Rα and SOCS1, together resulted in the T-bet^+^ OT-I cells being much more responsive to IL-15-dependent proliferation than were the T-bet-cells (Fig. 3C).

**Figure 4:**
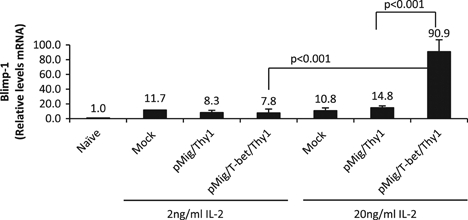
T-bet and regulation of IL-2-dependent Blimp-1 transcription. OT-I cells were transduced, purified and cultured with 2 ng/mL IL-2 or 20 ng/mL IL-2 in the presence of neutralising anti-IFN-γ, anti-IFN-γR1 and anti-IL-12 antibodies. After 48 h, RNA was extracted and assessed for Blimp-1 mRNA by qRT-PCR relative to CD3ɛ mRNA levels. All RNA samples were done in triplicate and the “No RT” controls were negative. Means and SD are shown from a representative of two independent experiments each with three treatment replicates. Briefly, *p*-values were determined by a one-way analysis of variance followed by Tukey's multiple comparison test for comparisons between all pairs of conditions.

As indicated in the Introduction section, the three phenotypes of the Blimp-1-dependent, “exhausted” CD8^+^ T cell generated during certain persistent viral infections, the T-bet-dependent, aberrantly differentiated CD8^+^ T cell occurring in the absence of CD4^+^ T-cell help, and the CD25high CD8^+^ T cell responding to a viral infection, have shared features. Accordingly, we used our *in vitro* system to determine whether T-bet and IL-2R signalling were involved in the expression of Blimp-1. Culture of the retrovirally transduced T-bet^+^ and T-bet^−^ OT-I cells in the presence of 2 ng/mL IL-2 did not induce the expression of Blimp-1. However, if culture was carried out with 20 ng IL-2/mL, which would be sufficient to saturate all IL-2R, even those lacking the IL-2Rα subunit, Blimp-1 expression increased six-fold in the OT-I cells expressing ectopic T-bet (Fig. 4). This change in Blimp-1 expression occurred with no apparent increase in T-bet expression above that mediated by the pMig/T-bet/Thy1 retroviral vector (Supporting Information Fig. 1). Thus, T-bet links IL-2R signalling to the expression of Blimp-1.

## Discussion

This study has resolved two questions regarding T-bet: whether its role in promoting the acquisition of effector functions by the CD8^+^ T cell is dependent on the cytokines that regulate its expression, and how it contributes to the terminal differentiation of the CD8^+^ T cell. Introducing ectopic T-bet into the CD8^+^ T cell was the most direct means of addressing these questions, but depended on having *in vitro* conditions that promoted the cell cycling of OT-I cells, which is necessary for retroviral transduction, but did not induce expression of endogenous T-bet. We have found that it is difficult, if not impossible, to purify populations of apparently naïve CD8^+^ T cells that are devoid of IFN-γ-producing capability when stimulated by TCR ligands. Thus, although such naïve cell preparations may be contaminated with only a minor population of IFN-γ-producing T cells, this source of IFN-γ will convert naïve T cells into a T-bet-expressing population that would obscure the effects of ectopically introduced T-bet. We were also concerned that splenocytes, the source of CD8^+^ T cells for these studies, may contain IL-12-producing cells that would promote the expression of T-bet. By including neutralising antibodies to these two cytokines, IFN-γ and IL-12, we were able to generate replicating CD8^+^ T cells that did not express endogenous T-bet after transduction with the control pMig retrovirus, and have an experimental system that could evaluate the effects of this transcription factor.

The capacity of CD8^+^ T cells expressing ectopic T-bet at physiological levels to acquire the effector functions of producing IFN-γ [17] and expressing FasL [19] indicates that these known effects of this transcription factor occur independently of the cytokines that normally induce its expression. T-bet expression alone also accounted for down-regulation of CD127 and SOCS-1 and up-regulation of CD122 and IL-15Rα, thereby switching the dependence for survival signals from IL-7 to IL-15. These findings are consistent with reports that T-bet expression selects against the differentiation of central memory CD8^+^ T cells [6, 7], which are characterized by the expression of CD127.

T-bet expression alone, however, did not induce the transcription of Blimp-1. Therefore, the development of the terminally differentiated CD8^+^ T cell, as represented by the expression of Blimp-1, can be separated from the acquisition of effector function, as has been recently emphasized by following the fate of cells that had expressed granzyme B during a viral infection [20]. Nevertheless, T-bet is linked to Blimp-1 in that it enables a high level of IL-2R signalling to induce Blimp-1. These observations may explain the findings of *in vivo* studies that sustained IL-2R signalling, T-bet and Blimp-1 each cause a similar phenotype of impaired proliferation and high Blimp-1 expression in CD8^+^ T cells that has been associated with the “exhausted” [15], “unhelped” [7] or “terminally differentiated” [8, 9] state. Each of these stages of development may be a manifestation of Blimp-1 expression, which augments effector function but is antithetical to continued participation of the CD8^+^ T cell in vigorous and rapid clonal expansion [13, 14]. Thus, T-bet alone in the presence of a low level of IL-2 signalling suffices for the acquisition of effector functions, but the requirements for T-bet to promote terminal differentiation are more stringent, perhaps because this developmental step may be accompanied by loss of expression of Bmi-1 and the occurrence of replicative senescence [21].

## Materials and methods

### Mice

C57BL/6 mice were purchased from Charles River UK Limited (Margate, UK). OT-I RAG^−/−^ mice were a generous gift from C. Reis e Sousa (Cancer Research UK London Research Institute, UK). Mice were housed under specific pathogen-free conditions at the University of Cambridge. For all animals, housing and procedures were carried out in accordance to the UK Home Office guidelines.

### Purification and stimulation of primary CD8^+^ T cells

Naïve CD8^+^ OT-I T cells were purified using negative selection with the CD8a^+^ T-cell Isolation Kit (Miltenyi Biotec) followed by positive selection with CD62L microbeads (Miltenyi Biotec). Transduced cells were purified by staining with anti-Thy1 APC antibody (clone HIS51, eBioscience) followed by positive selection with anti-APC microbeads (Miltenyi Biotec). Cells were stimulated with anti-CD3e clone 145-2C11 (eBioscience), either platebound (0.5 μg/mL), or with the OT-I TCR-specific antigen, SIINFEKL peptide (10 nM) (Peptides International) [22], in the presence of mouse IL-2 (2 ng/mL) (R&D Systems). Where indicated cell cultures were continued in IL-2 (2–20 ng/mL) or IL-15 (2–20 ng/mL). In conditions where neutralisation of IL-12, IFN-γ or Fas-FasL signalling was indicated, anti-FasL (clone MFL3) at 10–20 μg/mL, anti-IL-12 (clone C17.8) at 5 μg/mL, anti-IFN-γ (clone XMG1.2) at 5 μg/mL or anti-IFN-γR1 (R&D Systems) at 5 μg/mL were added.

### Flow cytometry

Intracellular staining for IFN-γ was performed using the Cytofix/Cytoperm kit (BD Biosciences) after stimulation with SIINFEKL peptide (10 nM) for 4 h. Annexin V and 7-AAD staining (Annexin V Apoptosis Detection Kit, BD Pharmingen) were performed according to the manufacturer's instructions. Antibodies used were anti-CD8a (53-6.7), anti-CD44 (IM7), anti-CD62L (MEL-14), anti-CD3e (145-2C11), anti-IFN-γ (XMG1.2), anti-CD127 (A7R34), anti-CD122 (5H4), anti-KLRG1 (2F1), anti-CD90.1 (HIS51), anti-IL-15Rα (FAB551F) and anti-FasL (MFL3) (all eBioscience).

### Viruses

The retroviral vector, pMig and the pCL-ECO packaging construct have been described previously [23]. Full-length T-bet cDNA was inserted into the pMig retroviral vector employing XhoI and EcoRI restriction enzyme sites, which had been modified by inserting Thy1 instead of EGFP downstream of the internal ribosome entry site (IRES) sequence. Moloney murine leukaemia virus expressing rat Thy1 alone (pMig-IRES-Thy1) or with T-bet (pMIG-T-bet-IRES-Thy1) was produced by transiently transfecting HEK293 cells at 90% confluency with an equimolar ratio of pMIG and the packaging construct, pCL-ECO with lipofectamine 2000 (Invitrogen). Primary CD8^+^ T cells were transduced 24 h after activation in the presence of IL-2, anti-IFN-γ, anti-IFN-γR1, anti-IL-12 and 6 μg/mL protamine sulphate (Sigma).

### Quantitative RT-PCR

Primers and TaqMan probes for the detection of murine Blimp1 (Mm00476128_m1), CD3ɛ (Mm00599683_m1), FasL (Mm00438864_m1), T-bet (Mm00450960_m1) and SOCS-1 (Mm00782550_s1) were purchased as Assays-on-Demand from Applied Biosystems.

### Western blot analysis

The antibodies used for immunoblot analysis of T-bet expression were mouse anti-T-bet antibody [4B10] (Santa Cruz Biotechnology), goat anti-mouse IgG_1_-HRP (Santa Cruz Biotechnology), rabbit anti-β-actin (Abcam), goat anti-rabbit IgG-HRP (Santa Cruz Biotechnology). Blots were developed with SuperSignal West Pico Chemiluminescent Substrate (Pierce) and membranes were stripped using Restore Western Blot Stripping Buffer (Pierce).

### MHV-68 infection

The murine gammaherpes virus expressing OVA, MHV-68, was a generous gift from Professor Philip Stevenson (Division of Virology, Department of Pathology, University of Cambridge, Cambridge, UK) [24]. Virus stocks were grown and titered on BHK-21 cells as has been described previously [25]. C57BL/6 mice were infected intranasally with 2×10^4^ pfu of MHV-68. Splenocytes were either depleted of B cells with anti-CD19 microbreads (Miltenyi Biotec), and CD8^+^ CD44^lo^, CD44^hi^ KLRG1^hi^ and CD44^hi^ KLRG1^lo^ were sorted (Daco MoFlo) after staining with appropriate monoclonal antibodies.

### Statistical analysis

All statistical analyses were performed using the Graphpad Prism 5 program and *p*-values <0.05 were considered significant. For comparisons between three or more conditions, *p*-values were determined by a one-way analysis of variance followed by Tukey's multiple comparison test for comparisons between all pairs of conditions. For comparisons between two conditions, *p*-values were determined by two-tailed paired or unpaired Student's *t*-tests.

## Abbreviations

Blimp-1: B lymphocyte-induced maturation protein 1
FasL: Fas ligand
IL-2R: IL-2 receptor
IL-15R: IL-15 receptor
IRES: internal ribosome entry site
KLRG1: killer cell lectin-like receptor G1
MHV-68: murine gammaherpesvirus 68
T-bet: T-box expressed in T cells or Tbx21
