# Supplementary material for: T-bet-mediated differentiation of the activated CD8+ T cell
Source: Eur J Immunol. 2010 Nov 19;41(1):60–6. doi: 10.1002/eji.201040873 (PMC3130140; doi:10.1002/eji.201040873)
Supplement: Supplementary file 1 [file eji0041-0060-SD1.pdf]

European Journal of Immunology

Supporting Information

for

DOI 10.1002/eji.201040873

T-bet-mediated differentiation of the activated CD8^{+} T cell

Crystal J. J. Yeo and Douglas T. Fearon

© 2010 WILEY-VCH Verlag GmbH & Co. KGaA, Weinheim

Supplementary Figure 1

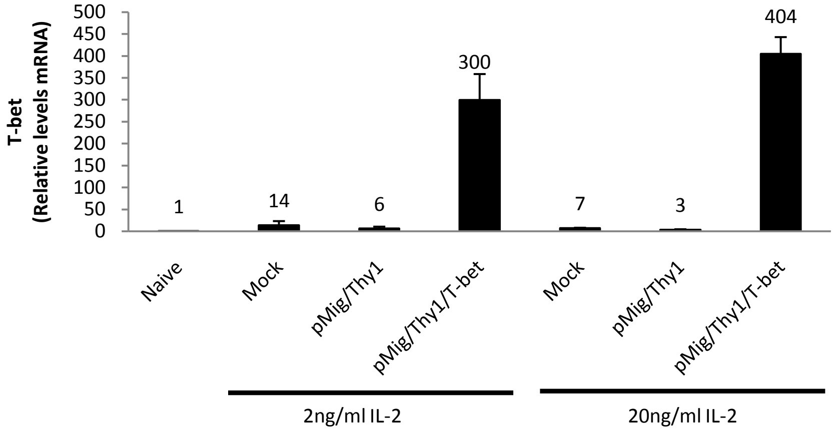

S1: IL-2 and T-bet expression
OT-I cells were transduced, purified, and cultured with 2ng/ml IL-2 or 20ng/ml IL-2 in the presence of neutralising anti-IFN-γ, anti-IFN-γR1 and anti-IL-12 antibodies. After 48 hours, RNA was extracted and assessed for T-bet mRNA by qRT-PCR relative to CD3epsilon mRNA levels. All RNA samples were done in triplicate and the "No RT" controls were negative. Cumulative means and SD are shown from 3 independent experiments each with 3 treatment replicates.
